# Supplementary material for: Bayesian inference of agent-based models: a tool for studying kidney branching morphogenesis
Source: J Math Biol. 2018 Feb 1;76(7):1673–97. doi: 10.1007/s00285-018-1208-z (PMC5906521; doi:10.1007/s00285-018-1208-z)
Supplement: Supplementary file 1 — Supplementary material 1 (pdf 618 KB) [file 285_2018_1208_MOESM1_ESM.pdf]

Bayesian inference of agent-based models: a tool for studying kidney branching morphogenesis

Ben Lambert[1,*,†], Adam L. MacLean[2,3,*], Alexander G. Fletcher[4,5], Alexander N. Combes[6,7], Melissa H. Little[7,8], Helen M. Byrne[2]
[1]Department of Zoology, University of Oxford, Oxford, UK
[2]Mathematical Institute, University of Oxford, Andrew Wiles Building, Woodstock Road, Oxford, UK
[3]Current address: Department of Mathematics, University of California, Irvine, Irvine CA, USA
[4]School of Mathematics & Statistics, University of Sheffield, Hicks Building, Hounsfield Road, Sheffield, S3 7RH, UK
[5]Bateson Centre, University of Sheffield, Firth Court, Western Bank, Sheffield, S10 2TN, UK
[6]Department of Anatomy & Neuroscience, University of Melbourne, Melbourne 3010 VIC, Australia
[7]Murdoch Childrens Research Institute, Flemington Rd, Parkville, Melbourne, 3052 VIC, Australia
[8]Department of Paediatrics, The University of Melbourne, Melbourne, 3010 VIC, Australia *These authors contributed equally.
[†]email: ben.c.lambert@gmail.com

1 Appendix

In this section we present results of an analysis conducted to assess the sensitivity of branching to the initial distribution of epithelial cells. We use images from two other experiments presented in Watanabe & Costantini (2004) to determine the shape of the initial bulk of epithelium that we use in simulations. We then perform the same simulations as described in the main text (see Table 1). These also reveal that GDNF-stimulated proliferation coupled with anisotropic cell division (and chemotaxis) are needed to recapitulate *ex vivo* branching.

Similarly Figs. S3 and S4 (analogous to Fig. 6 in the main text) show how branching depends on a finely-tuned GDNF proliferation switch for simulations initialised with the data from the other two explant experiments in Watanabe & Costantini (2004).

**Parameter**	**Description**	**All experiments**	**2,3**	**4,5A**	**4,5B**	**4,5C**	**4,5D**	**4,5E**
L	domain width and height (cell diameters)	400	-	-	-	-	-	-
d_g	non-dimensional diffusion coefficient	0.006	-	-	-	-	-	-
g_∞	non-dimensional GDNF at boundaries	1	-	-	-	-	-	-
p_{move}	probability of selecting move vs division	0.3 (varied in AABC)	0.3	0.3	0.3	0.3	0.3	0.3
c_1	GDNF-stimulated division switch parameter	-40 to 10	-25	10	-25	10	-25	10
c_2	GDNF-stimulated division switch parameter	0 to 400	120	0	120	0	120	0
β_m	chemotaxis GDNF sensitivity parameter	0 to 200	200	0	0	200	200	0
β_{cd}	ACD GDNF sensitivity parameter	0 to 200	200	0	0	0	0	200
		**4,5F**	**4,5G**	**4,5H**	**6A**	**6B**	**6C**	**7**
p_{move}	probability of selecting move vs division	0.3	0.3	0.3	0.3	0.3	0.3	0 to 1
c_1	GDNF-stimulated division switch parameter	-25	10	-25	-25	-25	-25	-40 to -20
c_2	GDNF-stimulated division switch parameter	120	0	120	400	120	20	40 to 280
β_m	chemotaxis GDNF sensitivity parameter	0	200	200	200	200	200	200
β_{cd}	ACD GDNF sensitivity parameter	200	200	200	200	200	200	200

Table S1: Summary of dimensionless parameter values used in each simulation. "ACD" indicates "anisotropic cell division".

References

Watanabe, Tomoko, & Costantini, Frank D. 2004. Real-time analysis of ureteric bud branching morphogenesis in vitro. *Developmental Biology*, **271**(1), 98–108.

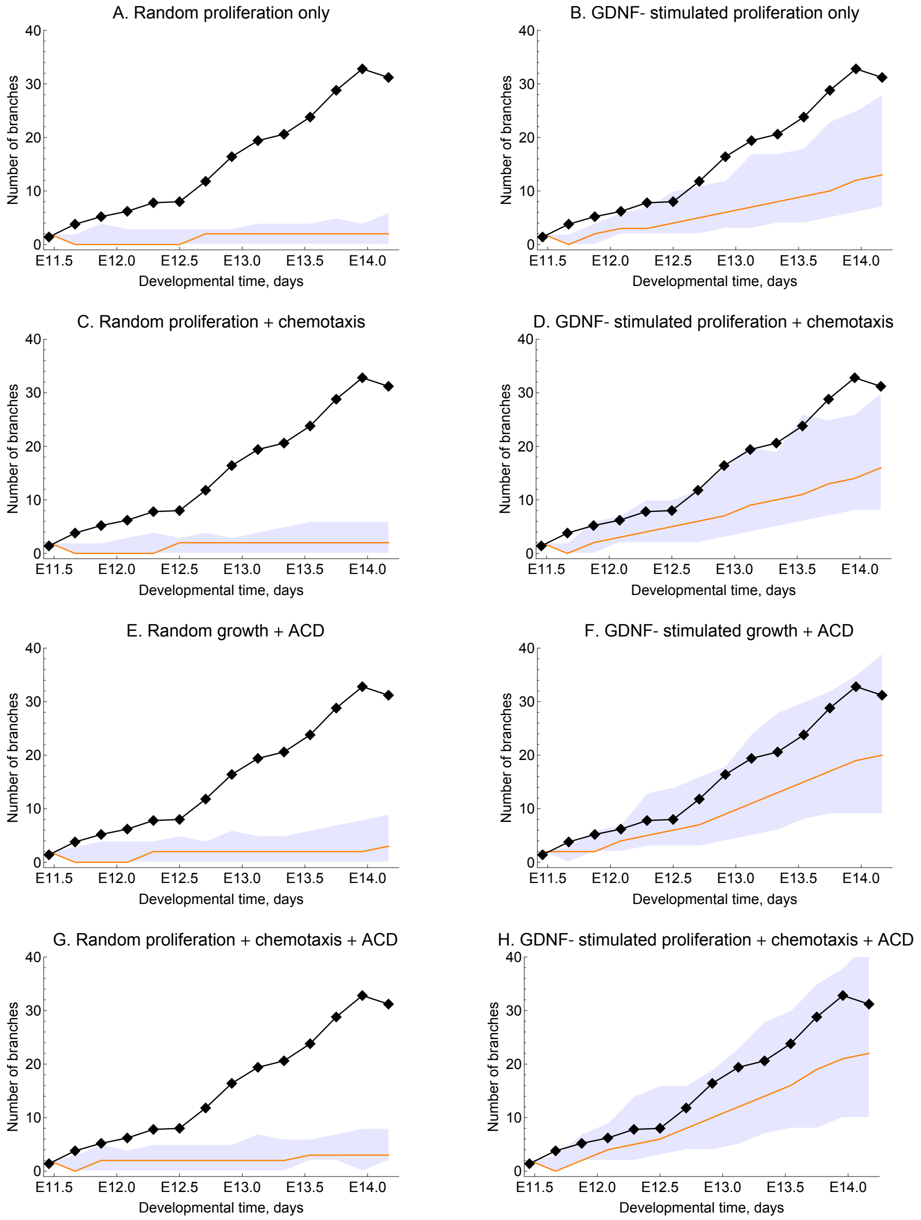

Figure S1: The effect of different GDNF signalling mechanisms on explant branching for another explant experiment. In each panel the black line and points represent the evolution of branches from an explant experiment in (Watanabe & Costantini, 2004); the orange line represents the mean branching observed by model simulation (n= 200) and the shaded region indicates the 95% confidence interval. “ACD” indicates “anisotropic cell division”. The parameter values used in each case are the same as for Fig. 4 in the main text. Note: these parameter ranges do not represent fitted parameter values, they are merely representative of the ranges of values that were used for simulation.

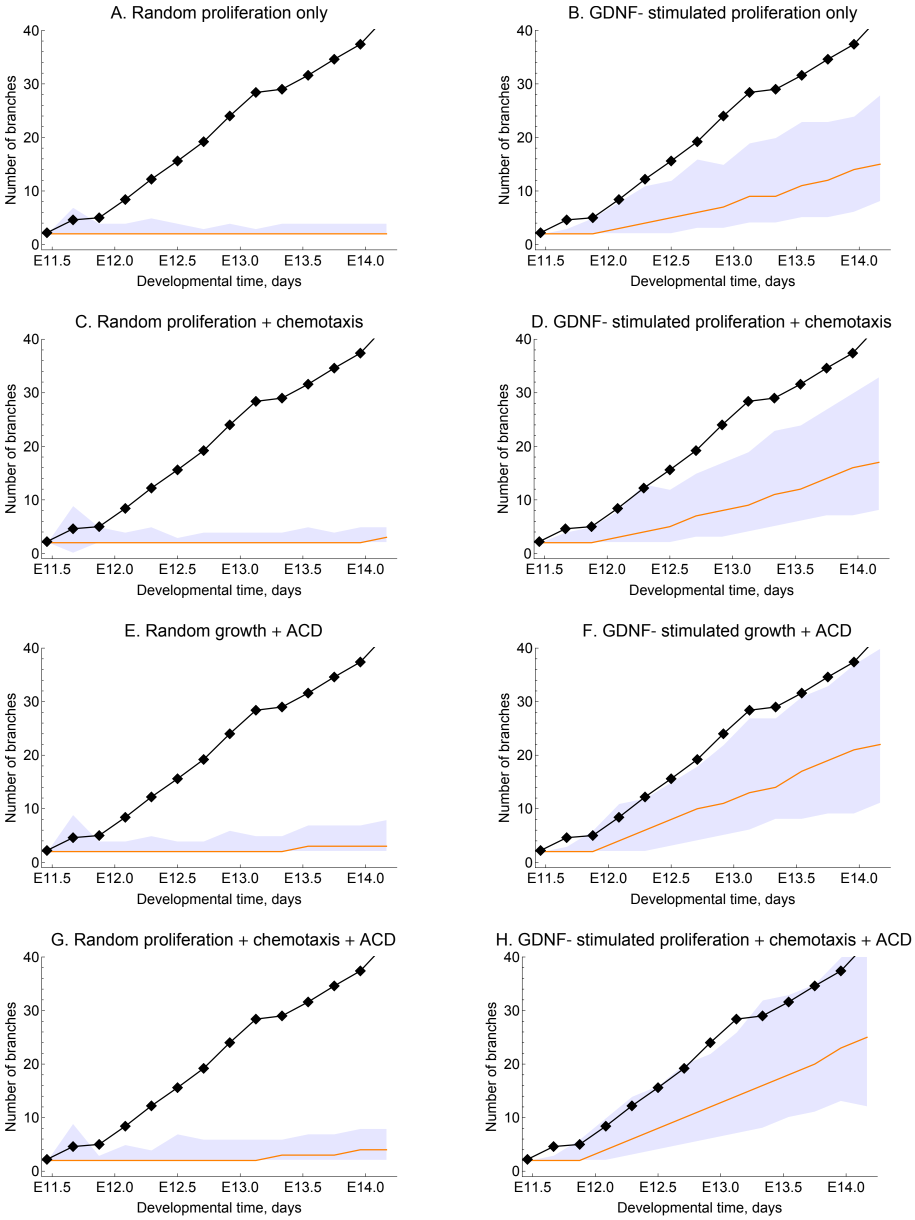

Figure S2: The effect of different GDNF signalling mechanisms on explant branching for a further explant experiment. In each panel the black line and points represent the evolution of branches from an explant experiment in (Watanabe & Costantini, 2004); the orange line represents the mean branching observed by model simulation (n= 200) and the shaded region indicates the 95% confidence interval. "ACD" indicates "anisotropic cell division". The parameter values used in each case are the same as for Fig. 4 in the main text.

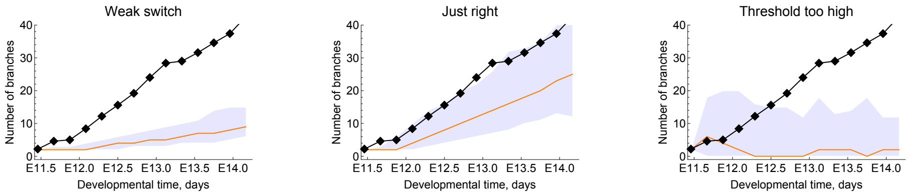

Figure S3: The effect of a GDNF-mediated proliferation growth switch on simulated explant branching for the same explant as in figure S1. In each panel the black line and points represent the evolution of branches from an explant experiment in (Watanabe & Costantini, 2004); the orange line represents the mean branching observed by model simulation (n= 200) and the shaded region indicates the 95% confidence interval. The parameter values used in each case are the same as for Fig. 6 in the main text.

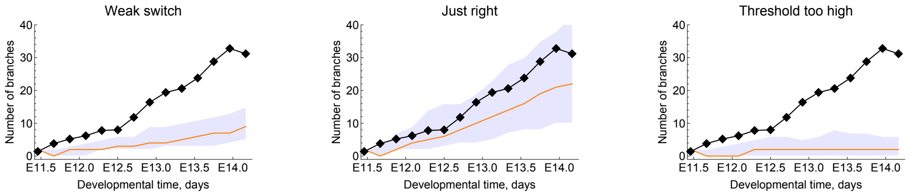

Figure S4: The effect of a GDNF-mediated proliferation growth switch on simulated explant branching for the same explant as in figure S2. In each panel the black line and points represent the evolution of branches from an explant experiment in (Watanabe & Costantini, 2004); the orange line represents the mean branching observed by model simulation (n= 200) and the shaded region indicates the 95% confidence interval. The parameter values used in each case are the same as for Fig. 6 in the main text.
